# Editorial: Epigenetics as a Deep Intimate Dialogue between Host and Symbionts

**DOI:** 10.3389/fgene.2016.00007

**Published:** 2016-02-09

**Authors:** Ilaria Negri, Eva Jablonka

**Affiliations:** ^1^Koiné - Environmental Consulting S.n.c.Parma, Italy; ^2^The Cohn Institute for the History and Philosophy of Science and Ideas, Tel-Aviv UniversityTel-Aviv, Israel

**Keywords:** epigenetics, holobiont, host-symbiont crosstalk, pathogen, DNA methylation, histone modifications, chromatin re-modeling, genome immunity

## Host-symbiont epigenetic crosstalk: A “*koiné* language” that enables communication between different species

In ancient Greek, the term *koiné* refers to a common language, which spread in the Eastern Mediterranean region and the Near East following the conquests of Alexander the Great. In the 4th century BC, *koiné* quickly became a shared language that enabled people speaking different dialects to communicate with each other. Indeed, effective communication is always necessary for overcoming “barriers” between individuals and groups, tightening relationships, and enabling new ways of coping with a changing world. This is the case not only for interactions involving cultural relations. Different living entities communicate in many ways, with the most intimate and often the most long-lasting communication being the formation of mutually beneficial, obligatory symbiotic associations. As Lynn Margulis forcefully argued, symbiotic relations are found among virtually all living organisms, and were critical for the origin and diversification of the eukaryotes (Sagan, 1967; Margulis, 1971; Guerrero et al., 2013). Since the partners in symbiotic relationships frequently belong to different kingdoms, and the intimacy may be so deep that the Symbionts reside in the tissues and/or cells of the host, their shared “language” should be a basic—and ancient—form of communication. Such effective communication blurs the boundaries between different living entities, giving rise to a single biomolecular network, a “holobiont” with a “hologenome” (Zilber-Rosenberg and Rosenberg, 2008; Bordenstein and Theis, 2015), thus problematizing the conventional notion of individuality. The series of papers presented in this topic-issue explore the fascinating developmental and evolutionary relationship between Symbionts and hosts, by focusing on the mediating epigenetic processes that enable the communication to be effective and robust at both the individual, the ecological, and the evolutionary time scales.

One of the currently most researched cases illustrating the productive cross-talk between Symbionts and hosts is the symbiosis between mammals and their gut microbiome. As Gilbert stresses, the birth of a mammal is not merely the origin of a new distinct (traditional) individual, but the onset of a new community. Already *in utero*, the fetus interacts with a network of symbiotic factors that is provided by the mother and, at birth, as it leaves the maternal symbiotic association system, it forms its own community which, although largely based on the legacy it gets from its mother, becomes rapidly adapted to its specific idiosyncratic conditions of life. Since the normal development and the thriving of the newborn depend on the symbiotic legacy it receives, it is the holobiont—the community of interacting species—that is the target of both developmental and natural selection. However, as Fridmann-Sirkis et al. illustrate, in extreme circumstances, when interactions fail, for example, because of acute stress to the microbiome, the host adapts by making adaptive changes in its epigenome, which can be inherited by subsequent generations. In more normal conditions, the maternal legacy provides crucial developmental resources, and is the basis on which flexible, context-sensitive, modifications of these resources, that allow rapid adaptation to the normally dynamic and fluctuating conditions in which the new individual lives, are constructed.

Since the microbiome reacts and evolutionarily adapts much faster than the host, most host's physiological adaptations are likely to be initiated by evolutionary changes to the constitution of the microbiome. Soen analyzes the manner in which the changes in the constitution of the microbiome lead to the modification of the communication process among the species comprising the microbiome, as well as between the microbiome and the host; and shows how these changes enable the developmental construction of a new dynamic, adaptive equilibrium, involving mutually beneficial epigenetic modifications in the host that can be epigenetically inherited between host generations. Hence, adaptive evolutionary modifications in the rapidly evolving microbiome, that occur at the time scale of host's individual life, are followed by the epigenetic inheritance of the host's physiological adaptations, thus enabling adaptive adjustments of the host at the ecological time scale (Soen; see also Moran and Sloan, 2015).

Although many symbiotic associations play essential roles in host development, physiology, and health, some associations may be harmful for the host. In these cases, the “selfish” Symbiont (often referred to as “parasite”) must evade the host's immune responses, and this may occur through “misleading” the host, for example, by mimicking the host's signaling and control system for its own benefits. Some intracellular pathogenic bacteria, such as *Anaplasma phagocytophilum* a pathogen studied by Sinclair et al. encode eukaryotic-like proteins (e.g., the nucleomodulin AnkA) for subverting the host cells metabolism by recruiting chromatin-modifying enzymes or by altering the folding patterns of chromatin that bring distant regulatory regions together to coordinate control of transcriptional reprogramming. Other epigenetic factors are SET-domain containing proteins, which are known to modify chromatin structure in eukaryotic cells. Alvarez-Venegas shows that SET domain genes, which meditate host-symbiont epigenetic interactions, and that have been identified in several bacterial genomes on the basis of their similarity to the SET domains of eukaryotic histone methyltransferases, allow pathogens to inhibit transcriptional activation of host defense genes. Indeed, there is plenty of evidence supporting the role of epigenetic mechanisms (e.g., DNA methylation, mechanisms that re-model chromatin structure through histone modifications, and mechanisms underlying RNA interference) both in pathogen plasticity and pathogen-induced alterations of the host (Gómez-Díaz et al., 2012). One such example is the molecular pathogenesis of Epstein–Barr virus (which induces diverse lymphoid and epithelial malignancies) discussed by Niller et al. that is accompanied by epigenetic alterations of both the viral and the host genomes.

Some “parasites” promote genetic exchange with the host genome. According to many studies, the exchange of human and viral genes must have happened repeatedly during evolution, and this can account for the high degree of homology between many viral and human genes. Niller et al. point to the example of the thymidylate synthase gene in the genome of *Herpesvirus saimiri*, which has 70% amino acids homology to that of the human gene, and Shapiro reviews and discusses the already massive and still growing evidence showing that such invasions into a host genome through virally-transmitted genetic elements (transposons, retrotransposons, and genomic proviruses) rewire the hosts' genomic networks, rearrange the host's genome and exert new types of selection pressures on it. The evolution of epigenetic silencing strategies, that prevent mobile DNA from destroying the integrity of the host genome and that have epigenetic heritable effects, is one such genomic adaptation. Indeed, it has been argued that one of the main functions of epigenetic mechanisms is a form of “genome immunity,” promoting protection against foreign genetic elements. Sagy et al. suggest that this cellular immune function may explain the systematic failure of cloning some eukaryotic genomes in bacteria. For example, about 20% of the nematode *Caenorhabditis elegans* genome is not clonable in *Escherichia coli*, but can be cloned in yeast, a eukaryote. Interestingly, the bacterially unclonable sequences are enriched with repetitive DNA transposons and PIWI-interacting small RNAs (piRNAs). In worms and many other organisms (other animals and protists), piRNA-mediated RNA interference has a role in genome surveillance against foreign sequences. Sagy et al. therefore propose that piRNAs may act *in trans* to eliminate bacteria (*E. coli*), thus preventing its ability to be cloned within the bacterium. As they note, this role of piRNAs may have therapeutic applications, and could be used against pathogenic bacteria. Another potential epigenetic- medical intervention can be based on a genome-wide profiling of histone modifications combined with gene expression in the human malaria vector *Anopheles gambiae*. Targeting changes in histone modification and transcription of genes that regulate malaria transmission may provide, as Gómez-Díaz et al. point out, new type of malaria control. Deciphering the mechanisms underlying the molecular “dialogue” between hosts and symbionts therefore seems of major importance for many research areas including medicine, genetics, cell biology, zoology, microbiology, evolutionary biology, and ecology.

## Concluding remarks and perspectives

The papers published in this topic-issue, which focus on the role of epigenetic factors and mechanisms in the interactions among the different species comprising the holobiont, point to important research directions. Epigenetic mechanisms enable dynamic developmental communication, an epigenetic *koiné*, between hosts and symbionts at several different time scales. We are confident that the molecular-epigenetic technologies already available—and those that are rapidly being developed—will provide important insights into the evolution and development of the organisms on our planet, whose history and future are based on ongoing communication and interactions.

## Author contributions

All authors listed have made substantial, direct and intellectual contribution to the work, and approved it for publication.

### Conflict of interest statement

The authors declare that the research was conducted in the absence of any commercial or financial relationships that could be construed as a potential conflict of interest.
